# Impact of diabetes duration and degree of carotid artery stenosis on major adverse cardiovascular events: a single-center, retrospective, observational cohort study

**DOI:** 10.1186/s12933-017-0556-0

**Published:** 2017-06-06

**Authors:** Minsu Noh, Hyunwook Kwon, Chang Hee Jung, Sun U. Kwon, Min Seon Kim, Woo Je Lee, Joong Yeol Park, Youngjin Han, Hyangkyoung Kim, Tae-Won Kwon, Yong-Pil Cho

**Affiliations:** 1 Department of Surgery, University of Ulsan College of Medicine and Asan Medical Center, Asanbyeongwon-gil 86, Songpa-gu, Seoul, 05505 South Korea; 2 Department of Internal Medicine, University of Ulsan College of Medicine and Asan Medical Center, Asanbyeongwon-gil 86, Songpa-gu, Seoul, 05505 South Korea; 3 Department of Neurology, University of Ulsan College of Medicine and Asan Medical Center, Asanbyeongwon-gil 86, Songpa-gu, Seoul, 05505 South Korea; 4 Department of Surgery, Chung-Ang University Hospital, 102 Heukseok-ro, Dongjak-gu, Seoul, 06973 South Korea

**Keywords:** Cardiovascular diseases, Carotid artery stenosis, Diabetes mellitus

## Abstract

**Background:**

We aimed to investigate the impact of diabetes duration and carotid artery stenosis (CAS) on the occurrence of major adverse cardiovascular events (MACE) in patients with type 2 diabetes mellitus (T2DM) without clinical cardiovascular disease.

**Methods:**

A total of 2006 patients with T2DM, without clinical cardiovascular disease, aged >50 years, and who underwent baseline carotid Doppler ultrasound screening with regular follow-ups at the outpatient clinic of our diabetes center, were stratified into four subgroups according to diabetes duration and CAS degree. The primary outcomes included the occurrence of MACE, defined as fatal or nonfatal stroke and myocardial infarction, and all-cause mortality.

**Results:**

The difference in the MACE incidence was significantly greater in patients with a longer diabetes duration (≥10 years) and significant CAS (50–69% luminal narrowing) (p < 0.001). Analysis of individual MACE components indicated a trend towards an increased incidence of stroke (p < 0.001), parallel to a longer diabetes duration and significant CAS. In contrast, the risk of myocardial infarction was significantly higher in patients with a diabetes duration <10 years and significant CAS (p = 0.039). Multivariate regression analysis showed that patients with both a longer diabetes duration and significant CAS demonstrated additive and very high risks of MACE (hazard ratio [HR], 2.07; 95% confidence interval [CI] 1.17–3.66; p = 0.012) and stroke (HR, 3.38; 95% CI 1.54–7.44; p = 0.002).

**Conclusions:**

The risk of MACE is significantly greater in patients with T2DM, without clinical cardiovascular disease, who have both a longer diabetes duration and significant CAS, compared with those who have a shorter duration and/or nonsignificant CAS.

**Electronic supplementary material:**

The online version of this article (doi:10.1186/s12933-017-0556-0) contains supplementary material, which is available to authorized users.

## Background

The incidence of diabetes-related morbidity and mortality declined between 1990 and 2010 owing to improvements in medical treatment [1]. Despite the presence of certain controversial findings, diabetes duration is considered an important contributor to the progression of atherosclerotic cardiovascular disease (CVD); moreover, carotid atherosclerosis is linked to a higher risk of major adverse cardiovascular events (MACE) [2–8]. Although previous studies have assessed the association between diabetes duration and the risk of MACE [9–11], it is unclear whether there is a comparable increased risk in patients with type 2 diabetes mellitus (T2DM) without clinical CVD. Many population-based studies on the relationship of the carotid intima-media thickness and the presence of focal plaque with CVD have shown that Doppler ultrasound (DUS) screening of carotid artery stenosis (CAS) can identify potentially high-risk patients in whom subsequent MACE may occur [12, 13]. Nevertheless, it is unknown whether both a longer duration of diabetes and a higher degree of CAS could result in an increased risk for the subsequent occurrence of MACE, in patients with T2DM, without clinical CVD. Further, it is also uncertain whether carotid DUS screening could be a valuable tool that can assist in CVD risk stratification for the application of preventive strategies in these patients.

In the present study, we aimed to investigate the impact of diabetes duration and CAS on the subsequent occurrence of MACE in patients with T2DM, without clinical CVD, and to determine the risk factors associated with clinical outcomes in these patients.

## Methods

### Study design and population

In this single-center, retrospective, observational cohort study, we analyzed data extracted from the medical records of patients. The study protocol was approved by our hospital’s institutional review board, which waived the need for informed consent. A total of 3136 consecutive patients with T2DM aged >50 years, who first visited the outpatient clinic of our diabetes center between January 2009 and December 2011, regardless of their diabetes duration, and underwent baseline carotid DUS screening with regular follow-ups were screened for inclusion in this study. The exclusion criteria were evidences of CVD including cerebral infarction, myocardial infarction (MI), angina, heart failure, and valvular heart disease; history of carotid or coronary revascularization; and severe CAS (≥70% stenosis based on the North American Symptomatic Carotid Surgery Trial [NASCET] criteria) on baseline DUS requiring carotid revascularization. Patients who were lost to follow-up were also excluded from the analysis.

### Clinical evaluation and definition of risk factor variables

The diagnosis of T2DM was based on the plasma glucose criteria, defined as either (i) a fasting plasma glucose level of ≥126 mg/dL, (ii) a 2-h plasma glucose level of ≥200 mg/dL after a 75-g oral glucose tolerance test, or (iii) a glycated hemoglobin level of ≥6.5% [14]. T2DM was confirmed by a result above the diagnostic threshold in two out of the three above-mentioned tests. In the absence of unequivocal hyperglycemia, results were confirmed by means of repeat testing. Patients who indicated the use of antidiabetic medications (oral hypoglycemia agents and/or insulin) on a self-report questionnaire were deemed to have T2DM. Diabetes onset was defined as the time when any of the above criteria was first met. Information on diabetes onset was obtained during patient interviews or by accessing medical records. Diabetes duration was estimated as the difference between the current age and the age at diabetes onset, and was classified as shorter (<10 years) or longer (≥10 years). DUS was performed by experienced radiologists in our diabetes center. The degree of CAS was estimated on the basis of peak systolic velocity (PSV) and end-diastolic velocity (EDV) values recorded from within the most stenotic segment in addition to luminal narrowing based on the NASCET [15]. Significant CAS was defined as 50–69% narrowing of the diameter of the common carotid artery, carotid bifurcation, or internal carotid artery, determined through analysis of the PSV in the range of 125–230 cm/s and EDV in the range of 40–100 cm/s [15]. The patients were categorized into four groups according to the diabetes duration and CAS degree: shorter diabetes duration and nonsignificant CAS (group 1), longer diabetes duration and nonsignificant CAS (group 2), shorter diabetes duration and significant CAS (group 3), and longer diabetes duration and significant CAS (group 4).

Body mass index was defined as weight (kg) divided by height squared (m^2^). Hypertension was defined as the use of antihypertensive medications or a systolic and/or diastolic blood pressure >140 and/or 90 mmHg, respectively (average of two readings taken by the examining physician). Dyslipidemia was defined as the use of lipid-lowering medications, a fasting total serum cholesterol level of >200 mg/dL, a low-density lipoprotein cholesterol level of >120 mg/dL, a high-density lipoprotein cholesterol level of <40 mg/dL, or a triglyceride level of >150 mg/dL [16]. Chronic kidney disease was defined as an estimated glomerular filtration rate of <60 mL/min/1.73/m^2^, which was assessed by using the modification of diet in renal disease formula [17].

### Outcomes of interest and follow-up

The primary outcomes included the occurrence of MACE, defined as fatal or nonfatal stroke and MI, and all-cause mortality. Only the first event of each outcome was included in the analysis.

Stroke was defined as an acute neurological event with focal symptoms and signs lasting for at least 24 h that were consistent with focal cerebral ischemia. Strokes were categorized as major or minor. A major stroke was present even after 7 days and increased the National Institutes of Health Stroke Scale (NIHSS) score of the patient by ≥4 points. A minor stroke was a transient ischemic attack, amaurosis fugax, or a stroke that completely resolved within 7 days and increased the NIHSS score of the patient by ≤3 points. We included only ischemic strokes in the analysis. MI was defined as any increase in creatine kinase–myocardial band or cardiac troponin I above the upper limit of the reference range, with either chest pain, symptoms consistent with ischemia, or electrocardiographic evidence of ischemia (i.e., new ST segment depression or elevation, or >1 mm elevation in two or more contiguous leads) during follow-up.

Follow-up visits with laboratory evaluations were scheduled at approximately 6-month intervals, and all medication adjustments were made by the patient’s health-care provider at our diabetes center. Follow-up cardiac enzyme levels, 12-lead electrocardiograms, and carotid DUS were performed depending on individual CVD risk factors. Follow-up ceased with the occurrence of MACE. Risk factors of interest, clinical characteristics, and long-term clinical outcomes for all patients were recorded in an Excel database (Microsoft Corp., Redmond, WA, USA) and analyzed retrospectively.

### Statistical analysis

Categorical variables are reported as frequencies or percentages, and continuous variables as means and standard deviations or medians and interquartile ranges (IQRs). Categorical variables were compared by using Chi square tests with the Bonferroni correction for multiple comparisons, whereas continuous variables were compared by using one-way analysis of variance with Tukey’s test or Kruskal–Wallis test for multiple comparisons, as appropriate. The cumulative probability of events was estimated with Kaplan–Meier analysis and compared with that estimated with the log-rank test. Univariate and multivariate analyses of the association of clinical variables with each outcome were conducted with Cox proportional hazards modeling, by using the event of interest and period from study enrollment to the date of the event or last follow-up as the outcome. Univariate Cox proportional hazard regression models were fitted to calculate hazard ratios (HRs) with 95% confidence intervals (CIs) to estimate the association of clinical variables with the occurrence of MACE. Variables with a *p* value of <0.1 on univariate analysis were included in multivariate Cox proportional hazard regression models, by using the backward elimination method. A p value of <0.05 was considered statistically significant. Statistical analyses were performed with SPSS version 21.0 (SSPS Inc., Chicago, IL, USA).

## Results

**Fig. 1:**
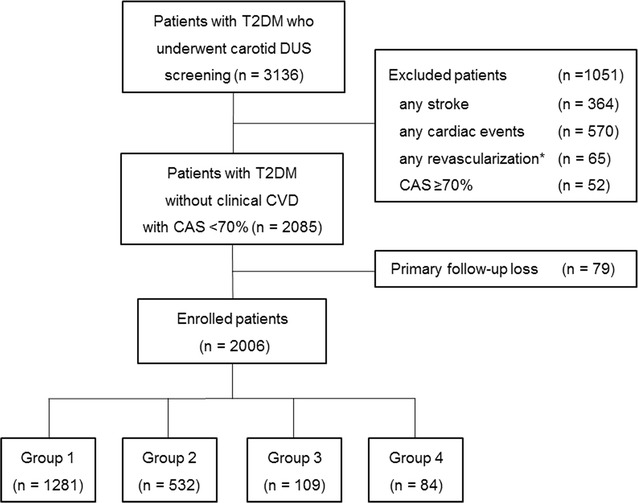
Flowchart of study inclusion. *CAS* carotid artery stenosis, *CVD* cardiovascular disease, *DUS* carotid Duplex ultrasound, *T2DM* type 2 diabetes mellitus. *Asterisk* history of carotid and/or coronary revascularization

Of the 3136 consecutive patients with T2DM who first visited the outpatient clinic of our diabetes center and were aged >50 years, we excluded 934 patients (29.8%) with prior CVD, 65 (2.1%) with a history of carotid or coronary revascularization, and 52 (1.7%) with severe CAS on baseline DUS requiring carotid revascularization. A further 79 patients (2.5%) who were lost to follow-up were also excluded from the analysis. The remaining 2006 patients (64.0%) without clinical CVD at baseline, and with a reported CAS of <70% on baseline DUS and regular follow-ups, were included in the analysis (Fig. 1). Eligible patients were stratified into four groups according to the diabetes duration and CAS degree as follows: group 1 (n = 1281, 63.9%), group 2 (n = 532, 26.5%), group 3 (n = 109, 5.4%), and group 4 (n = 84, 4.2%).

**Fig. 2:**
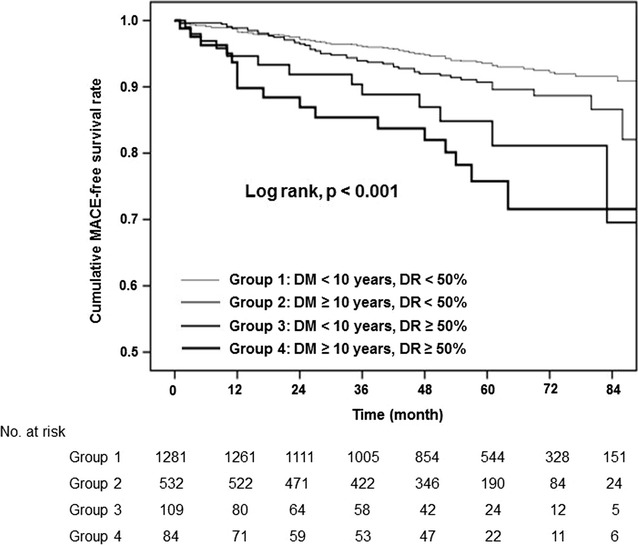
Kaplan–Meier analyses of the cumulative event-free rates. Cumulative event-free rates of composite MACE according to the diabetes duration and degree of carotid artery stenosis. *DM* diabetes mellitus, *DR* diameter reduction of the carotid artery

The baseline characteristics of the patients are presented in Table 1. During the mean follow-up period of 55.7 ± 21.2 months, the MACE incidence was found to be 5.9, 8.6, 11.9, and 20.2% in groups 1–4, respectively (Table 2). The difference was significantly greater in patients with a longer diabetes duration and significant CAS (p < 0.001). Analysis of the individual MACE components indicated a trend toward an increased incidence of stroke (p < 0.001), but not of all-cause mortality (p = 0.694), in parallel with a longer diabetes duration and the presence of significant CAS, whereas the risk of MI was significantly higher in patients with diabetes duration <10 years and significant CAS (group 3) (p = 0.039). Kaplan–Meier survival analysis showed that patients with a longer diabetes duration and significant CAS had a decreased MACE-free survival rate (p < 0.001), as compared with those with a shorter diabetes duration and nonsignificant CAS (Fig. 2; Additional file 1: Figure S1).

**Table 1:** Baseline characteristics of the study population stratified by diabetes duration and carotid artery stenosis

	Group 1	Group 2	Group 3	Group 4	p value
No. of patients	1281	532	109	84	
DM duration (years)	2.7 (0.5–7.0)	15.5 (13.0–20.0)	3.9 (2.0–8.0)	15.6 (13.0–21.0)	
Mean age (years)	60.2 ± 7.3	63.0 ± 7.8	65.5 ± 7.9	69.4 ± 7.3	<0.001
Male sex	872 (68.1)	330 (62.0)	93 (85.3)	56 (66.7)	<0.001
BMI (kg/m^2^)	25.2 ± 3.2	24.5 ± 3.1	24.5 ± 3.1	23.9 ± 3.8	<0.001
Risk factor					
Hypertension	608 (47.5)	290 (54.5)	76 (69.7)	55 (65.5)	<0.001
Dyslipidemia	506 (39.5)	191 (35.9)	32 (29.4)	30 (35.7)	0.119
CKD	25 (2.0)	36 (6.8)	6 (5.5)	9 (10.7)	<0.001
Current smoker	248 (19.4)	68 (12.8)	18 (16.5)	10 (11.9)	0.004
Medication					
Antiplatelet	339 (26.5)	207 (38.9)	65 (59.6)	40 (47.6)	<0.001
Statin	548 (42.8)	262 (49.2)	52 (47.7)	45 (53.6)	0.027
Antihypertensive	531 (41.5)	238 (44.7)	69 (63.3)	47 (56.0)	<0.001
Insulin	97 (7.6)	137 (25.8)	12 (11.0)	20 (23.8)	<0.001
Laboratory data					
HbA1c (%)	7.0 ± 1.2	7.5 ± 1.2	7.2 ± 1.4	7.5 ± 1.3	<0.001
Cr (mg/dL)	0.9 ± 0.5	1.1 ± 1.0	1.0 ± 0.7	1.2 ± 1.0	<0.001
eGFR (mL/min/1.73/m^2^)	74.5 (60.0–87.0)	63.4 (60.0–80.0)	61.8 (60.0–79.0)	60.9 (57.0–75.5)	<0.001
Total cholesterol (mg/dL)	177.3 ± 38.9	164.0 ± 34.4	165.2 ± 39.7	170.2 ± 50.4	<0.001
HDL (mg/dL)	50.2 ± 13.1	51.1 ± 14.3	45.8 ± 12.9	49.2 ± 13.2	0.002
LDL (mg/dL)	105.9 ± 33.0	94.8 ± 29.9	95.9 ± 32.1	99.1 ± 3 7.3	<0.001
TG (mg/dL)	121.3 (90.0–169.0)	112.5 (82.0–154.0)	119.0 (84.0–185.0)	105.3 (78.0–148.5)	0.045

Continuous data are presented as mean ± standard deviation or medians (interquartile ranges); categorical data are given as numbers (%)

*BMI* body mass index, *CKD* chronic kidney disease, *Cr* creatinine, *DM* diabetes mellitus, *eGFR* estimated glomerular filtration rate, *HbA1c* glycated hemoglobin, *HDL* high-density lipoprotein cholesterol, *LDL* low-density lipoprotein cholesterol, *TG* triglyceride

**Table 2:** Major adverse cardiovascular events (MACE) in the study patients stratified according to diabetes duration and degree of carotid artery stenosis

	Group 1	Group 2	Group 3	Group 4	p value
Number of patients	1281 (63.9)	532 (26.5)	109 (5.4)	84 (4.2)	
MACE	76 (5.9)	46 (8.6)	13 (11.9)	17 (20.2)	<0.001
Any stroke	23 (1.8)	19 (3.6)	4 (3.7)	12 (14.3)	<0.001
Major stroke	13 (1.0)	7 (1.3)	2 (1.8)	6 (7.1)	<0.001
Minor stroke	10 (0.8)	12 (2.3)	2 (1.8)	6 (7.1)	<0.001
MI	39 (3.0)	20 (3.8)	9 (8.3)	4 (4.8)	0.039
Death^a^	14 (1.1)	7 (1.3)	0 (0.0)	1 (1.2)	0.694
Follow-up (months)	56.5 ± 21.9	54.7 ± 18.7	52.4 ± 23.1	55.0 ± 21.3	0.143

Values are presented as numbers (%)

*MI* myocardial infarction

^a^All-cause mortality, except for deaths of cardiovascular causes

When the combined effects of diabetes duration and CAS were analyzed on multivariate Cox proportional hazard regression analysis, after adjusting for confounding variables, patients with both a longer diabetes duration and significant CAS demonstrated additive and very high risks for MACE occurrence (HR, 2.07; 95% CI 1.17–3.66; p = 0.012; Table 3) and stroke (HR, 3.38; 95% CI 1.54–7.44; p = 0.002; Additional file 2: Table S1). Male sex (HR, 1.98; 95% CI 1.10–3.57; p = 0.024), hypertension (HR, 1.66; 95% CI 1.03–2.70; p = 0.039), and poor glycemic control, as reflected by the glycated hemoglobin level (HR, 1.27; 95% CI 1.09–1.48; p = 0.002), were all significantly associated with MI (Additional file 2: Table S2), whereas age (HR, 1.05; 95% CI 1.00–1.11; p = 0.039), chronic kidney disease (HR, 4.29; 95% CI 1.41–13.04; p = 0.010), and insulin use (HR, 2.54; 95% CI 1.01–6.38; p = 0.047) were associated with all-cause mortality (Additional file 2: Table S3). Both a longer diabetes duration and significant CAS were not associated with increased risks of MI (HR, 1.60; 95% CI 0.57–4.50; p = 0.374) and all-cause mortality (not applicable). Multivariate analyses that evaluated the respective effects of diabetes duration and CAS on MI indicated that CAS was significantly associated with the risk of MI (p = 0.007), whereas diabetes duration was not associated with any such risk (p = 0.908) (data not shown).

**Table 3:** Factors associated with the occurrence of major adverse cardiovascular events

	Univariate analysis		Multivariate analysis	
	HR (95% CI)	p value	HR (95% CI)	p value
Age	1.06 (1.04–1.08)	<0.001	1.04 (1.02–1.06)	<0.001
Male sex	1.27 (0.89–1.81)	0.188	NA	NA
Body mass index	0.99 (0.95–1.04)	0.775	NA	NA
Hypertension	2.33 (1.65–3.28)	<0.001	1.82 (1.28–2.61)	0.001
CKD	4.29 (2.68–6.86)	<0.001	2.71 (1.66–4.44)	<0.001
Smoking	1.09 (0.73–1.63)	0.667	NA	NA
Antiplatelet use	1.53 (1.11–2.11)	0.009	NA	NA
Statin use	0.92 (0.67–1.26)	0.589	NA	NA
Insulin use	1.62 (1.08–2.43)	0.019	NA	NA
HbA1c value	1.22 (1.10–1.36)	<0.001	1.21 (1.08–1.35)	0.001
Creatinine level	1.24 (1.09–1.41)	0.001	NA	NA
Group 1	Reference			
Group 2	1.52 (1.06–2.19)	0.023	1.07 (0.74–1.56)	0.712
Group 3	2.86 (1.59–5.15)	<0.001	1.93 (1.06–3.51)	0.032
Group 4	4.11 (2.43–6.95)	<0.001	2.07 (1.17–3.66)	0.012

*CI* confidence interval, *CKD* chronic kidney disease, *HbA1c* glycated hemoglobin, *HR* hazard ratio, *NA* not applicable

## Discussion

The major finding of the present study on patients with T2DM without clinical CVD was that the cumulative risk of MACE is significantly higher in those with a longer diabetes duration and significant CAS. Although previous studies have described the risk of MACE conferred by the presence and severity of CAS [4, 18], the association between the diabetes duration and MACE occurrence remains controversial; in fact, certain studies have indicated an association, whereas others have not [19, 20]. Furthermore, no reports definitely document the cumulative effects of both a longer diabetes duration and significant CAS on long-term clinical outcomes in patients with T2DM, without clinical CVD. The present study evaluated the impact of both diabetes duration and CAS degree, and found that a longer diabetes duration and significant CAS had additive and much higher risks for MACE and stroke, although the diabetes duration did not affect the risk of MI.

The risk of MI was significantly higher in patients with diabetes duration <10 years and significant CAS (group 3), which primarily included those with male sex and a higher prevalence of hypertension. Considering that male sex and hypertension were significantly associated with an increased risk of MI in our analysis (Additional file 2: Table S2), and that the number of patients in groups 3 and 4 was small, we speculated that these baseline differences may have affected the MI incidence between the study populations. Additional large cohort studies are required to evaluate the association between diabetes duration and MI in patients with T2DM without clinical CVD. Our current results also showed that the rate of all-cause mortality was markedly low, as compared with that in other population-based studies, because our study included only the first event of each outcome and most of the deaths from cardiovascular causes were excluded from all-cause mortality.

CVD is the leading cause of morbidity and mortality worldwide, and its prevention is less costly than the treatment of its complications. Hence, the identification of subclinical disease during the asymptomatic phase has emerged as a public health and economic objective [21]. Cardiovascular risk assessment is generally recommended based on conventional risk factors. However, the predictive ability is only moderate, and a significant percentage of patients, irrespective of the conventional risk factors, present with subclinical atherosclerosis on DUS imaging along with coronary artery calcification [22, 23]. Although DUS screening of the carotid artery can identify potentially high-risk patients in whom subsequent MACE may occur, and some studies have assessed the association between CAS and MACE risk [24–28], the impact of both diabetes duration and CAS on the MACE risk of patients with T2DM without clinical CVD remains unclear. Furthermore, although previous studies had described the risk of MACE conferred by the presence and severity of CAS in blacks and whites, there are very few reports available for Asians [4]. According to the data from the Department of Measurement and Health Information of the World Health Organization, there is a significant difference in the risk of MACE between different racial–ethnic groups; stroke is more prominent than MI in Asian general populations compared with Western general populations [29, 30]. Therefore, our findings may serve as novel data that could aid in the optimal surveillance and management of MACE risk in Asian patients with T2DM without clinical CVD.

Glucose intolerance and diabetes are risk factors for stroke and MI in Asia, as in Western countries [31]. Although Asian populations are presently less obese than Western populations, the recent Westernization of dietary habits has resulted in increasing body mass index in most Asian countries. Moreover, the prevalence of glucose intolerance and diabetes may increase further. A large-scale meta-analysis project, the Asia Pacific Cohort Studies Collaboration, showed that the HRs of diabetes for ischemic stroke and MI are similar for both Asian and Western countries [32]. The prevalence of diabetes has been increasing throughout Asia, and the speed of increase is much faster than in Western countries [29]. Trends in increased prevalence of diabetes and higher risks of diabetes-related MACE reported by several population-based studies showed the importance of the development of primary prevention programs for this vulnerable and fastest-growing higher-risk population in Asia. In our study, the degree of CAS, as detected by using carotid DUS screening, is a simple and noninvasive marker of subclinical atherosclerosis associated with an increased risk of MACE in patients with T2DM, without clinical CVD. Furthermore, DUS screening could be a valuable tool that can assist in cardiovascular risk stratification for the application of preventive strategies.

This study has some notable limitations. First, our findings may have been subject to selection and information bias owing to the retrospective study design; hence, the incidence of MACE may have been underestimated, and the number of excluded patients was considerable. Moreover, despite efforts to optimize blood pressure, glucose, and lipid control during the management of patients at our diabetes center, we sometimes failed to achieve management goals defined by the annually updated Standards of Medical Care in Diabetes by the American Diabetes Association [33, 34]. We could not analyze microvascular complications. Furthermore, we could not evaluate carotid plaque characteristics such as ulceration, surface irregularities, and morphologic composition, because our study cohort received DUS as a routine screening for cardiovascular risk factors at our diabetes center. For routine screening of subclinical atherosclerosis of the carotid artery, the availability, ease of measurement, and cost will be of major importance in choosing the imaging methodology [4]. Imaging of carotid plaque and measurement of CAS degree is a simple noninvasive and cost-effective method. Although some methods, such as measurements of plaque area and volume, may be better in risk prediction than our method [35–37], these imaging methodologies are more complex, time consuming, expensive, and not readily available [4]. There could be intra- and inter-observer variability affecting our analyses because the reliability of DUS examination highly depends on the expertise of the examiner and the knowledge and experience of the interpreting physician. Carotid DUS was performed by accredited radiologists in our diabetes center, and all images were reviewed by two experienced vascular surgeons. Second, our study cohort was preselected for being otherwise healthy patients with T2DM without clinical CVD. Therefore, our results may not be applicable to the general population with diabetes, and we could not compare our findings with those of other population-based studies [38, 39]. Furthermore, the cohort consists of only Korean Asians and may not be representative of other Asians or other racial–ethnic minority groups; our findings should be interpreted with caution with respect to different racial–ethnic groups because the environmental and genetic factors may differ among various racial–ethnic groups, and there may be racial–ethnic differences in the prevalence of MACE in patients with T2DM. Third, we calculated the diabetes duration at baseline by using the participant self-reported age at diabetes onset, which may have been inaccurate owing to the lag between the time of disease onset and diagnosis. There may have been several years of dysglycemia, which also increases cardiovascular risk. Furthermore, our analysis between diabetes duration and MACE was complicated by the fact that a longer diabetes duration is associated with older age, and the residual confounding effects of this association cannot be excluded. Furthermore, our current findings were obtained at a single center, resulting in a small sample size, which may limit the overall relevance of our results. Lastly, the mean follow-up duration may not have been sufficient for the occurrence of MACE.

## Conclusions

The findings of the present study on patients with T2DM without clinical CVD indicated that there is a significantly higher risk of MACE in patients with a longer diabetes duration and significant CAS. In fact, both diabetes duration and CAS have additive and very high risks for MACE and stroke.

## Additional files

**Additional file 1: Figure S1.** Kaplan–Meier analyses of the cumulative event-free rates. Cumulative event-free rates of (**A**) stroke, (**B**) myocardial infarction, and (**C**) all-cause mortality according to the diabetes duration and degree of carotid artery stenosis. DM, diabetes mellitus; DR, diameter reduction of the carotid artery.

**Additional file 2: Table S1.** Factors associated with the occurrence of stroke. **Table S2.** Factors associated with the occurrence of myocardial infarction. **Table S3.** Factors associated with the occurrence of all-cause mortality.

## Abbreviations

CVD: cardiovascular disease
CAS: carotid artery stenosis
CI: confidence interval
DUS: Doppler ultrasound
EDV: end-diastolic velocity
HR: hazard ratio
MACE: major adverse cardiovascular events
MI: myocardial infarction
PSV: peak systolic velocity
T2DM: type 2 diabetes mellitus
