# Supplementary material for: Impact of diabetes duration and degree of carotid artery stenosis on major adverse cardiovascular events: a single-center, retrospective, observational cohort study
Source: Cardiovasc Diabetol. 2017 Jun 6;16:74. doi: 10.1186/s12933-017-0556-0 (PMC5461631; doi:10.1186/s12933-017-0556-0)
Supplement: Supplementary file 1 — Additional file 1: Figure S1. Kaplan–Meier analyses of the cumulative event-free rates. Cumulative event-free rates of (A) stroke, (B) myocardial infarction, and (C) all-cause mortality according to the diabetes duration and degree of carotid artery stenosis. DM, diabetes mellitus; DR, diameter reduction of the carotid artery. [file 12933_2017_556_MOESM1_ESM.docx]

**
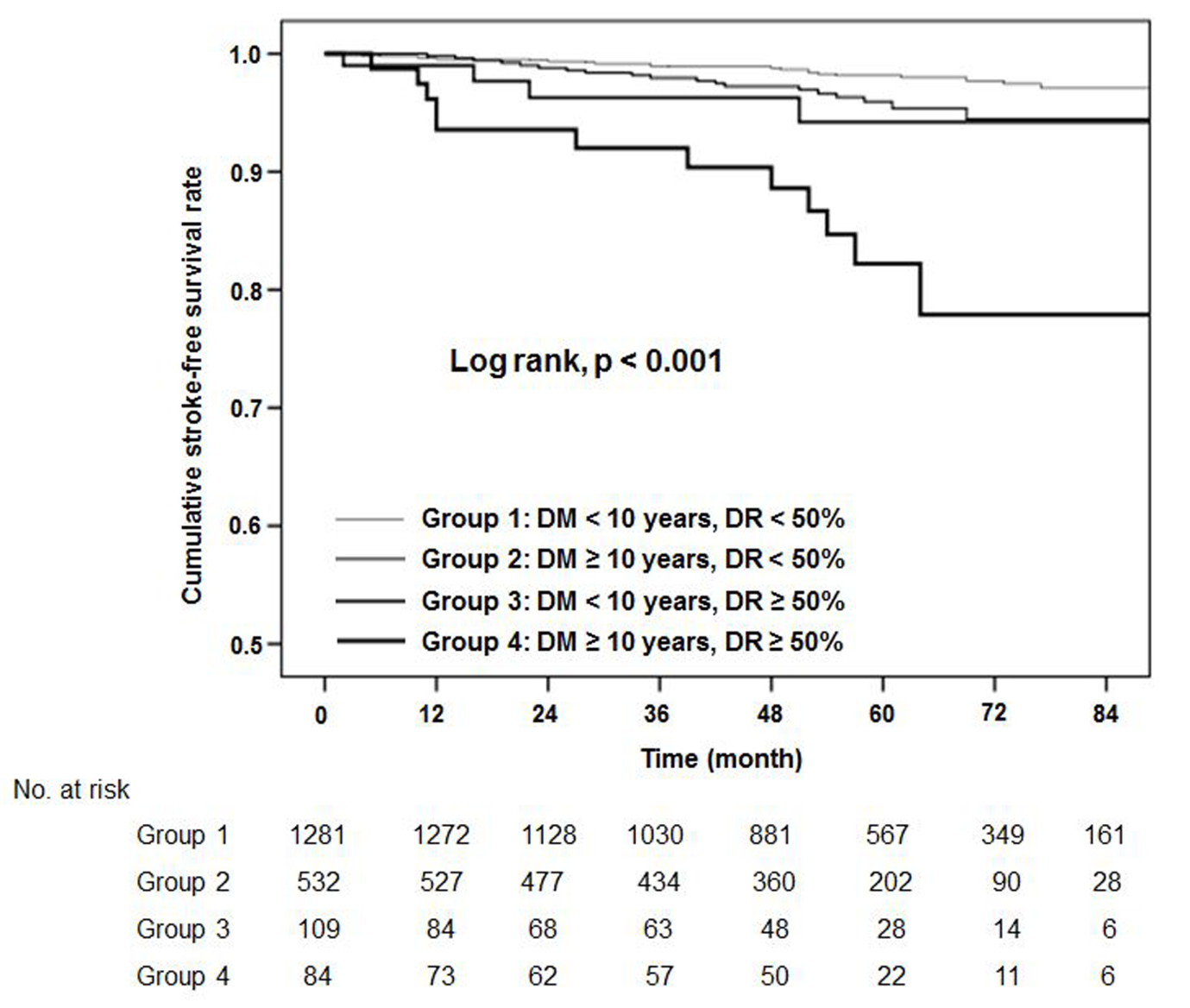
**

**Figure S1A**

**
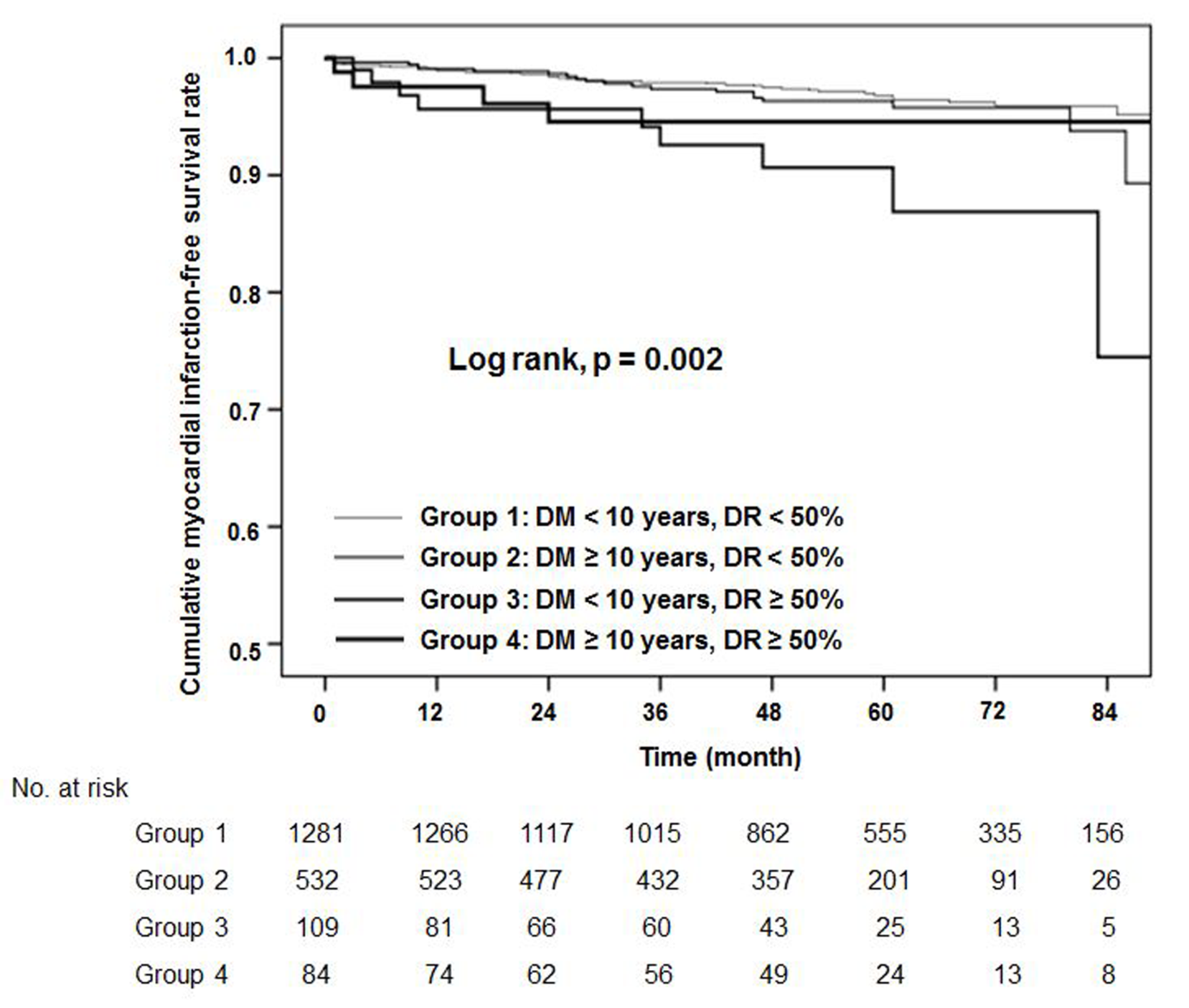
**

**Figure S1B**

**
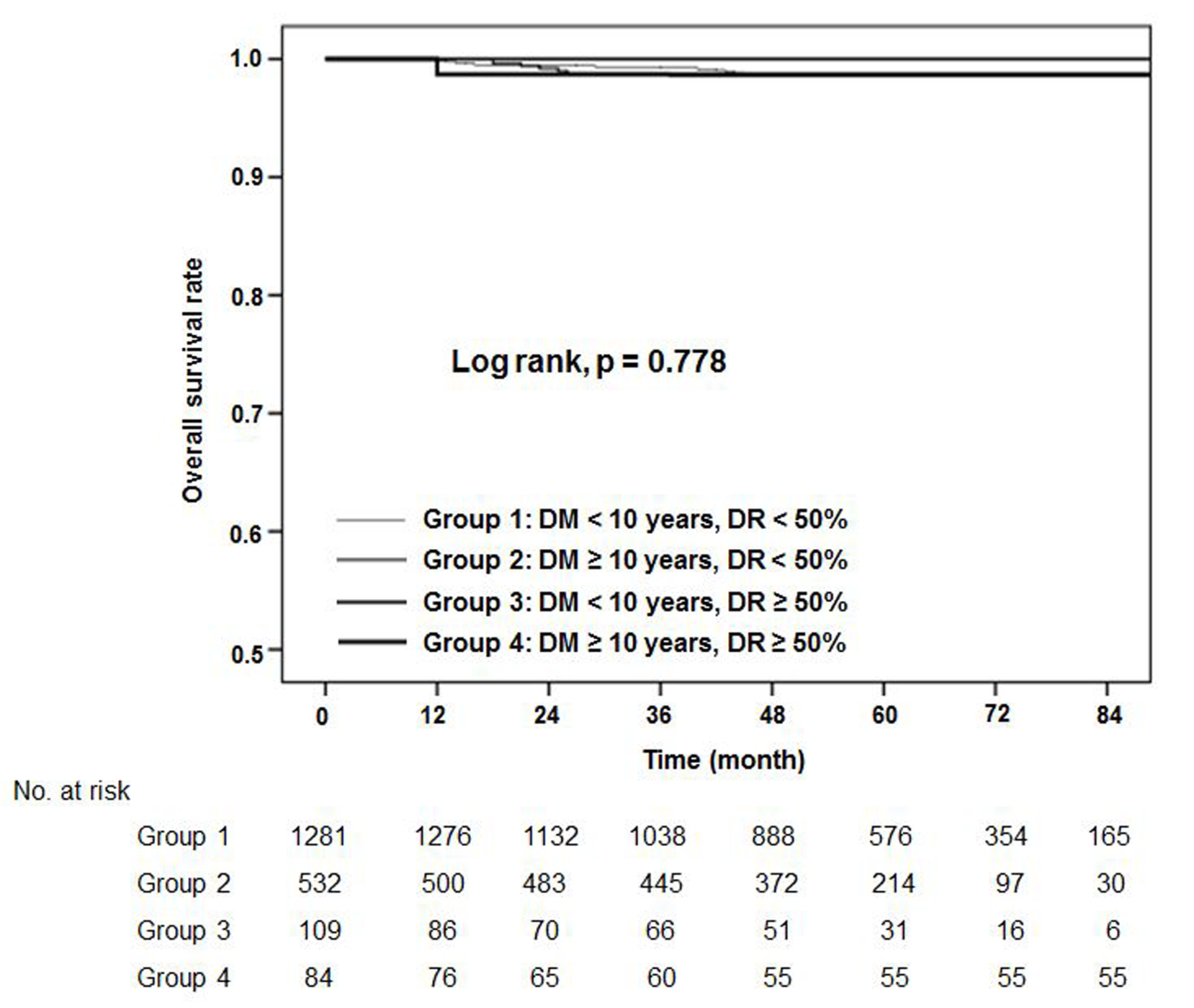
**

**Figure S1C**

**Legends**

**Supplementary Figure S1** Kaplan–Meier analyses of the cumulative event-free rates

Cumulative event-free rates of **(A)** stroke, **(B)** myocardial infarction, and **(C)** all-cause mortality according to the diabetes duration and degree of carotid artery stenosis.

DM, diabetes mellitus; DR, diameter reduction of the carotid artery.
