# Supplementary material for: Impact of diabetes duration and degree of carotid artery stenosis on major adverse cardiovascular events: a single-center, retrospective, observational cohort study
Source: Cardiovasc Diabetol. 2017 Jun 6;16:74. doi: 10.1186/s12933-017-0556-0 (PMC5461631; doi:10.1186/s12933-017-0556-0)
Supplement: Supplementary file 2 — Additional file 2: Table S1. Factors associated with the occurrence of stroke. Table S2. Factors associated with the occurrence of myocardial infarction. Table S3. Factors associated with the occurrence of all-cause mortality. [file 12933_2017_556_MOESM2_ESM.docx]

**Supplementary Table S1** Factors associated with the occurrence of stroke

Univariate analysis Multivariate analysis

HR (95% CI) p-Value HR (95% CI) p-Value

Age 1.11 (1.07–1.14) <0.001 1.08 (1.04–1.11) <0.001

Male sex 0.87 (0.51–1.50) 0.625 NA NA

BMI 0.99 (0.92–1.08) 0.845 NA NA

Hypertension 4.29 (2.23–8.27) <0.001 2.83 (1.44–5.58) 0.003

CKD 6.87 (3.56–13.26) <0.001 3.36 (1.67–6.76) 0.001

Smoking 1.29 (0.70–2.40) 0.415 NA NA

Antiplatelet 1.65 (0.98–2.77) 0.060 NA NA

Statin 0.73 (0.43– .25) 0.250 NA NA

Insulin 2.08 (1.12–3.86) 0.021 NA NA

HbA1c 1.25 (1.06–1.48) 0.008 1.24 (1.04–1.49) 0.017

Creatinine 1.28 (1.05–1.55) 0.013 NA NA

Group 1 Reference

Group 2 2.10 (1.14–3.85) 0.017 1.29 (0.69–2.42) 0.427

Group 3 2.96 (1.02–8.55) 0.046 1.59 (0.54–4.66) 0.398

Group 4 9.77 (4.86–19.66) <0.001 3.38 (1.54–7.44) 0.002

BMI, body mass index; CI, confidence interval; CKD, chronic kidney disease; HbA1c, glycated hemoglobin; HR, hazard ratio; NA, not applicable.

**Supplementary Table S2** Factors associated with the occurrence of myocardial infarction

Univariate analysis Multivariate analysis

HR (95% CI) p-Value HR (95% CI) p-Value

Age 1.01 (0.99–1.04) 0.349 NA NA

Male sex 1.93 (1.08–3.47) 0.027 1.98 (1.10–3.57) 0.024

BMI 1.04 (0.97–1.11) 0.328 NA NA

Hypertension 1.65 (1.03–2.66) 0.039 1.66 (1.03–2.70) 0.039

CKD 2.04 (0.82–5.07) 0.123 NA NA

Smoking 1.28 (0.74–2.24) 0.382 NA NA

Antiplatelet 1.53 (0.96–2.45) 0.073 NA NA

Statin 1.09 (0.68–1.73) 0.726 NA NA

Insulin 0.89 (0.43–1.85) 0.751 NA NA

HbA1c 1.25 (1.08–1.45) 0.003 1.27 (1.09–1.48) 0.002

Creatinine 1.11 (0.85–1.46) 0.442 NA NA

Group 1 Reference

Group 2 1.30 (0.76–2.23) 0.341 1.12 (0.65–1.95) 0.679

Group 3 3.77 (1.83–7. 80) <0.001 3.04 (1.46–6.32) 0.003

Group 4 1.83 (0.65–5.12) 0.250 1.60 (0.57–4.50) 0.374

BMI, body mass index; CI, confidence interval; CKD, chronic kidney disease; HbA1c, glycated hemoglobin; HR, hazard ratio; NA, not applicable.

**Supplementary Table S3** Factors associated with the occurrence of all-cause mortality

Univariate analysis Multivariate analysis

HR (95% CI) p-Value HR (95% CI) p-Value

Age 1.06 (1.01–1.11) 0.030 1.05 (1.00–1.11) 0.039

Male sex 1.05 (0.43–2.58) 0.913 NA NA

BMI 0.91 (0.79–1.05) 0.194 NA NA

Hypertension 2.09 (0.85–5.14) 0.107 NA NA

CKD 5.96 (2.02–17.61) 0.001 4.29 (1.41–13.04) 0.010

Smoking 0.23 (0.30–1.67) 0.144 NA NA

Antiplatelet 1.23 (0.52–2.93) 0.642 NA NA

Statin 0.84 (0.36–1.97) 0.690 NA NA

Insulin 3.06 (1.25–7.51) 0.015 2.54 (1.01–6.38) 0.047

HbA1c 0.96 (0.68–1.36) 0.817 NA NA

Creatinine 1.40 (1.13–1.73) 0.002 NA NA

Group 1 Reference

Group 2 1.18 (0.48–2.92) 0.723 NA NA

Group 3 NA NA NA NA

Group 4 1.22 (0.16–9.29) 0.846 NA NA

BMI, body mass index; CI, confidence interval; CKD, chronic kidney disease; HbA1c, glycated hemoglobin; HR, hazard ratio; NA, not applicable.
